# Doubling of triazole resistance rates in invasive aspergillosis over a 10-year period, Belgium, 1 April 2022 to 31 March 2023

**DOI:** 10.2807/1560-7917.ES.2025.30.18.2400559

**Published:** 2025-05-08

**Authors:** Lize Cuypers, Robina Aerts, Otto Van de gaer, Lore Vinken, Rita Merckx, Veerle Gerils, Greetje Vande Velde, Agustin Reséndiz-Sharpe, Johan Maertens, Katrien Lagrou

**Affiliations:** 1 Department of Laboratory Medicine, National Reference Centre for Mycosis, University Hospitals Leuven, Leuven, Belgium; 2 Department of Microbiology, Immunology and Transplantation, Laboratory of Clinical Microbiology, KU Leuven, Leuven, Belgium; 3 Department of Internal Medicine, University Hospitals Leuven, Leuven, Belgium; 4 Department of Imaging and Pathology, Biomedical MRI unit, KU Leuven, Leuven, Belgium; 5 Department of Haematology, University Hospitals Leuven, Leuven, Belgium; * These authors contributed equally to this work and share first authorship.

**Keywords:** *Aspergillus fumigatus*, triazole resistance, national prospective surveillance, Belgium, CYP51A sequencing, empirical antifungal treatment

## Abstract

**Background:**

Dutch national treatment guidelines for fungal infections have been adapted based on surveillance findings of triazole resistance rates >10% in *Aspergillus* species isolates. In Belgium, nationwide resistance data have not been collected since 2011.

**Aim:**

Our objective was to evaluate changes in antifungal susceptibility among *Aspergillus* species isolates from patients with invasive aspergillosis.

**Methods:**

Laboratories across Belgium were invited to send all clinically relevant *Aspergillus* species isolates from patients diagnosed with invasive aspergillosis, collected between April 2022 and March 2023, to the National Reference Centre for Mycosis at UZ Leuven for identification and antifungal susceptibility testing.

**Results:**

Overall, 29 clinical laboratories contributed 309 isolates from 297 patients. Median patient age was 66 years (range: 6 months–96 years). Among isolates, 61% (189/309) were from male patients. At species level, *Aspergillus fumigatus* isolates predominated (278/309, 90%), with a 9.7% (27/278) triazole resistance rate, compared to the 4.6% rate found in 2011. Of 27 resistant isolates, successful *Cyp51A* sequencing of 26 showed 20 with the TR_34_/L98H resistance mechanism. Across the country, local *A. fumigatus* triazole resistance rates varied. Among provinces in the Flanders region, Antwerp had the highest resistance rate (15.4%: 10/65; p = 0.082), Flemish Brabant (6/48) also had a rate >10%, while Limburg (2/46) had the lowest rate.

**Conclusions:**

Geographical differences in *A. fumigatus* triazole resistance rates stress the importance of implementing broad prospective surveillance initiatives, not limited to one region or one hospital. In Belgium, triazole resistance rates have doubled over 10 years, nearly attaining the 10% threshold, warranting re-evaluation of local empirical antifungal treatment regimen decisions.

**Key public health message**

**What did you want to address in this study and why?**

Aspergillosis is an invasive disease caused by the fungus *Aspergillus*. When *Aspergillus* is resistant to antifungals, this challenges the disease treatment. In Belgium, the previous nationwide prospective surveillance study focusing on antifungal resistance in *Aspergillus* species isolates dates from more than 10 years ago. We wanted more recent resistance data from across the country to support potential updates of antifungal treatment guidelines.

**What have we learnt from this study?**

We observed that, among patients with invasive aspergillosis in Belgium, resistance rates in the species *Aspergillus fumigatus* have doubled over a 10-year period, from 4.6% in 2011 to 9.7% in 2022–2023. The threshold of 10%, previously used in the Netherlands to adapt Dutch national treatment guidelines for fungal infections, is almost reached in Belgium, warranting a re-evaluation of antifungal therapy regimens to potentially improve patient outcomes.

**What are the implications of your findings for public health?**

Next to the doubled resistance rate, geographical differences in Belgium were observed, with even rates > 10% in the two densely populated provinces of Antwerp and Flemish Brabant. This shows that sustained and long-term resistance surveillance data at country and provincial level are required to guide treatment decisions and that there is room to further improve the detection of antifungal resistance in clinical practice.

## Introduction

*Aspergillus* species produce billions of spores to ensure their survival and spread. In susceptible patients these fungi can cause severe disease, and globally incidence of invasive fungal infections has risen since the 2010s, partly due to the increased size of the population at risk [1].

According to current international guidelines (2016), triazole antifungals are recommended as first choice for prophylaxis and treatment of *Aspergillus* diseases [2]. While resistance to triazoles can gradually develop during antifungal therapy, more frequently, triazole-naïve patients become infected with a strain that gained resistance properties externally, as similar triazoles are both used in the environment and in the clinic [3-5]. Azole fungicide exposure in agriculture compromises the efficacy of medical azoles, making triazole resistance in *Aspergillus fumigatus* a One Health resistance threat [6]. Typically, resistance mechanisms involve changes in the *Cyp51A* gene’s promoter and coding regions, which result in tandem repeats (TR) of different lengths (34 or 46 base pairs) involving cytosine and thymine alternations in the promoter, and amino acid substitutions in the encoded protein. More specifically these are the TR_34_/L98H and TR_46_/Y121F/T289A mutations. Both are reported to be selected in the environment [4], with most isolates bearing these mutations displaying resistance to multiple azoles [7], considerably limiting treatment options. Triazole resistance is associated with an excess overall mortality in patients with invasive aspergillosis (IA) [8].

Since the first reports in 2007 of concerning increases in the prevalence of resistance to first-line therapies among *Aspergillus* species strains [9], triazole-resistant *A. fumigatus* has been observed frequently worldwide [10]. In Belgium, a 1-year prospective multicentre study was organised in 2011, which found an overall triazole resistance prevalence of 5.5%, with 4.6% specifically for IA [11]. Sub-analysis of isolates from haematology patients with culture-positive IA revealed a prevalence of 20% voriconazole resistance, associated with higher mortality rates [12]. Screening of nearly 2,500 *A. fumigatus* complex isolates between 2016 and 2020 at the National Reference Centre (NRC) for Mycosis at UZ Leuven, Belgium, showed a stable prevalence of 7.1% for triazole resistance [7].

The European Society of Clinical Microbiology and Infectious Diseases – European Confederation of Medical Mycology European Respiratory Society (ESCMID-ECMM-ERS) 2017 guidelines on management of *Aspergillus* disease recommend that in settings with environmental triazole resistance rate of >10%, first-line therapy with voriconazole plus an echinocandin or liposomal amphotericin B should be used [13]. Based on surveillance data showing increasing resistance rates for *Aspergillus* isolates, Dutch national treatment guidelines for fungal infections have been adapted [14]. For Belgium, nationwide prospective resistance data are lacking since 2011 [11], challenging the correct evaluation of changes in epidemiology and susceptibility of *Aspergillus* species isolates over time. 

This prospective study aimed to evaluate potential changes in epidemiology and susceptibility of clinical *Aspergillus* species isolates in Belgium, to advance knowledge on triazole resistance, consider updates of antifungal treatment guidelines, and improve patient outcomes.

## Methods

### Collection of isolates and statistical analyses

All Belgian clinical microbiological laboratories were invited to send *Aspergillus* species isolates cultured from clinical samples collected between 1 April 2022 and 31 March 2023 to the NRC for Mycosis at UZ Leuven.

The aim was to include in the study isolates that were clinically relevant, with classification as proven or probable IA disease following revised definitions from the European Organisation for Research and Treatment of Cancer/Mycoses Study Group Education and Research Consortium (EORTC/MSGERC) [15], as putative according to (modified) *Asp*ICU [16], as influenza-associated aspergillosis (IAPA) based on expert case definitions [17], or as COVID-19-associated aspergillosis (CAPA) according to the European Confederation of Medical Mycology and the International Society for Human and Animal Mycology (ECMM/ISHAM) criteria [18]. If classification within more than one definition was possible, it was based on the main driving disease. To prevent selection bias, laboratories were asked to send all clinically relevant isolates.

Isolates were sent accompanied by information on the participating centre, sampling date and site of isolation, individual patient demographic characteristics, such as age, sex (collected as male/female), as well as area of residence (postal code), underlying disease and antifungal treatment (initiated, prophylaxis or administered during the last year). Multiple isolates per patient could be enrolled when originating from multiple IA episodes or if multiple *Aspergillus* species were cultured. Isolates were grouped by provinces of origin, based on the postal code of the patients’ residence at the time of isolation of the fungus.

Data management was performed in RedCap, and all analyses were performed with the statistical software R studio (version 2024.09.0) [19]. Chi-square test, or Fisher test when appropriate, were used to analyse categorical variables, and Mann–Whitney U-test (or Kruskall–Wallis when > 2 groups) for continuous data; with a two-tailed significance level of p < 0.05.

### Identification, antifungal susceptibility testing and *Cyp51A* gene sequencing

All isolates were identified to species level by combination of microscopy and matrix-assisted laser desorption/ionisation time-of-flight (MALDI-TOF) mass spectrometry (Bruker Biotyper, Bruker Corporation, United States (US)) using the Mass Spectrometry Imaging (MSI) reference spectra database V2.0. In case of inconclusive identification, beta tubulin gene sequencing was performed [20]. Triazole resistance screening was performed for all *A. fumigatus* isolates using VIPCheck (MediaProducts, the Netherlands), followed by confirmation by the European Committee on Antimicrobial Susceptibility Testing (EUCAST) broth microdilution in case of growth [12]. Isolates were designated as triazole-resistant when minimum inhibitory concentrations (MIC)s of itraconazole, voriconazole, posaconazole, and/or isavuconazole (Sigma-Aldrich, US) were above EUCAST clinical breakpoints (v10.0, 2020). Triazole resistant *A. fumigatus* isolates were processed with *Cyp51A* gene sequencing [12]. In absence of EUCAST clinical breakpoints, cut-off values described in the literature, which are presented in Supplementary Table S1 [21], were used. Clinical breakpoints and cut-off values employed for the interpretation of amphotericin B susceptibility are also detailed in Supplementary Table S1.

### Detailed genotyping of resistant isolates

Genetic relatedness between *A. fumigatus* resistant isolates was determined as described earlier [22]. Genetic distance similarity and hierarchical relationship based on short tandem repeats (STR) between all resistant study isolates and 74 publicly available selected susceptible and resistant isolates (worldwide) from the AfumID-STR profile application were assessed using Rstudio (2024.09.0).

## Results

### Inclusion of 309 *Aspergillus* species isolates

During the course of 1 year, 29 clinical laboratories spread across Belgium (22 in Flanders, 5 in Wallonia, and 2 in Brussels) participated. *Aspergillus* species strains isolated from patients who did not meet the consensus definitions, were excluded, as well as multiple isolates from the same *Aspergillus* species and from the same disease episode per patient, resulting in the inclusion of 309 isolates from 297 patients, as shown in Supplementary Figure S1. For 12 patients, more than one isolate was included due to the occurrence of multiple aspergillosis episodes (4 cases) or when different *Aspergillus* species were isolated during the same episode (8 cases).

### Predominant inclusion of *Aspergillus fumigatus* isolates from Flanders

A description of the study cohort can be found in Table 1. Overall, 61.2% (189/309) of isolates were from male cases. For all patients in the cohort, the median age was 66 years (range: 6 months–96 years) and *Aspergillus* species were mainly isolated from respiratory specimens (97%; 301/309).

**Table 1:** Description of the final study cohort, based on *Aspergillus* species isolates derived from 297 invasive aspergillosis patients, Belgium, 1 April 2022–31 March 2023 (n = 309 *Aspergillus* species isolates)

Patient and isolate characteristics	Total isolates n = 309				p value^a^
	Susceptible isolates n = 272		Triazole-resistant isolates n = 37		
	Number	% (of 272)	Number	% (of 37)	
**Sex of patients from whom isolates were obtained**					
Female	103	37.9	17	45.9	0.444
Male	169	62.1	20	54.1	
**Age in years of patients**					
Median (IQR)	67 (14)		63 (16)		**0.033**
**Sample origin**					
UZ Leuven hospital	83	30.5	15	40.5	0.298
External laboratories	189	69.5	22	59.5	
**Isolation sample type**					
BALf	114	41.9	16	43.2	0.158
Bronchial aspirate	64	23.5	8	21.6	
Tracheal aspirate	20	7.4	0	0.0	
Sputum	62	22.8	8	21.6	
Pleural fluid	2	0.7	1	2.7	
Lung biopsy	4	1.5	2	5.4	
Other	6	2.2	2	5.4	
**Season cultured for 2022–2023**					
Spring	105	38.6	15	40.5	0.951
Summer	82	30.1	12	32.4	
Autumn	38	14.0	4	10.8	
Winter	47	17.3	6	16.2	

BALf:  bronchoalveolar lavage fluid; IQR:  interquartile range.

^a^ Bold font is used to show p values indicating statistical significance.

Grouping of isolates by provinces of origin showed a predominant inclusion of isolates from Flanders (90.6%, n = 280), compared with Wallonia (5.8%, n = 18) and Brussels (2.6%, n = 8). Three isolates originated from patients living abroad. The number of isolates was overall evenly distributed across Flanders, with the highest number from the province of Antwerp (23.9%, n = 74) (Table 2).

**Table 2:** *Aspergillus* species isolates, stratified by origin and antifungal susceptibility test results, Belgium, 1 April 2022–31 March 2023 (n = 309 *Aspergillus* species isolates)

Isolates’ provenance	Total cohort n = 309				p value	Total isolates per region or per province and proportion resistant in such localities (%)	
	Susceptible isolates n = 272		Triazole-resistant isolates n = 37				
	Number	% (of 272)	Number	% (of 37)		Total	%
**Region in Belgium of patients or provenance from abroad**							
Flanders region	245	90.1	35	94.6	0.851	280	12.5
Brussels region	7	2.6	1	2.7		8	12.5
Wallonia region	17	6.3	1	2.7		18	5.6
Abroad	3	1.1	0	0.0		3	0.0
**Provinces with >45 isolates**							
Antwerp^*,**^	60	22.1	14	37.8	0.154	74	18.9
Limburg^**^	48	17.6	3	8.1		51	5.9
East Flanders	43	15.8	5	13.5		48	10.4
Flemish Brabant	47	17.3	9	24.3		56	16.1
West Flanders	47	17.3	4	10.8		51	7.8
**Provinces of patients in Belgium or provenance from abroad**							
Antwerp	60	22.4	14	37.8	0.354	74	18.9
Brussels	7	2.5	1	2.7		8	12.5
Hainault	2	0.7	1	2.7		3	33.3
Limburg	48	17.7	3	8.1		51	5.9
Liège	11	4.0	0	0.0		11	0.0
Luxembourg	2	0.7	0	0.0		2	0.0
Namur	2	0.7	0	0.0		2	0.0
East Flanders	43	15.9	5	13.5		48	10.4
Flemish Brabant	47	17.3	9	24.3		56	16.1
West Flanders	47	17.0	4	10.8		51	7.8
Abroad	3	1.1	0	0.0		3	0.0

^*^ p = 0.082: Antwerp vs other Flemish provinces (Limburg, East Flanders, Flemish Brabant and West Flanders).

^**^ p = 0.060: Antwerp vs Limburg provinces.

There was a predominance of *A. fumigatus* complex (91.3%, n = 282), followed by the complexes *A. niger* (3.9%, n = 12), *A. flavus* (3.2%, n = 10), *A. nidulans* (0.6%, n = 2), *A. terreus* (0.3%, n = 1), *A. circumdatus* (0.3%, n = 1) and *A. ustus* (0.3%, n = 1) (Table 3).

**Table 3:** *Aspergillus* species complexes identified among isolates from 297 invasive aspergillosis patients, Belgium, 1 April 2022–31 March 2023 (n = 309 *Aspergillus* species isolates)

*Aspergillus* species complex	Total cohort n = 309				p value**^a^**
	Susceptible isolates n = 272		Triazole-resistant isolates n = 37		
	Number	% (of 272)	Number	% (of 37)	
**Identified complexes and other *Aspergillus* species**					
*Aspergillus fumigatus* complex (n = 282; 91.3% of 309)	253	93.0	29	78.4	**0.001**
*Aspergillus niger* complex (n = 12; 3.9% of 309)	9	3.3	3	8.1	
*Aspergillus flavus* complex (n = 10; 3.2% of 309)	9	3.3	1	2.7	
*Aspergillus nidulans* complex (n = 2; 0.6% of 309)	1	0.4	1	2.7	
*Aspergillus terreus* complex (n = 1; 0.3% of 309)	0	0.0	1	2.7	
Other *Aspergillus* species (n = 2; 0.6% of 309)	0	0.0	2	5.4	
**Grouping of *Aspergillus species complexes***					
*Aspergillus fumigatus complex* (n = 282; 91.3% of 309)	253	93.0	29	78.4	**0.008**
Non-*Aspergillus fumigatus complex* (n = 27; 8.7% of 309)	19	7.0	8	21.6	

^a^ Bold font is used to show p values indicating statistical significance.

### Clinical characteristics of all invasive aspergillosis cases

Isolates from episodes were classified as proven IA (4.2%, 13/309), probable IA (53.4%, 165/309), putative IA (17.8%, 55/309), CAPA (17.5%, 54/309) and IAPA (7.1%, 22/309). A high number and variety of co-morbidities characterised the study population, with often multiple underlying diseases involved, yet the level of detail was frequently insufficient to evaluate their true impact. Haematological malignancy was observed as main underlying disease in less than 13% of patients (Table 4).

**Table 4:** *Aspergillus* species isolates from 297 invasive aspergillosis patients, according to clinical characteristics of respective patients, Belgium, 1 April 2022–31 March 2023 (n = 309 isolates)

Patient and isolate characteristics	Total cohort n = 309				p value^a^	Total isolates and proportion (%) resistant per disease	
	Susceptible isolates n = 272		Triazole-resistant isolates n = 37				
	Number	% (of 272)	Number	% (of 37)		Total	%
**Classification of invasive aspergillosis**							
Proven invasive aspergillosis^*, **^	10	3.7	3	8.1	0.602	13	4.2
Probable invasive aspergillosis^**^	144	52.9	21	56.8		165	53.4
IAPA^***^	21	7.7	1	2.7		22	7.1
CAPA^***^	48	17.6	6	16.2		54	17.5
Putative invasive aspergillosis	49	18.0	6	16.2		55	17.8
**Main underlying disease**							
Haematologic malignancy	31	11.4	9	24.3	NA^b^	40	12.9
Non-haematologic malignancy	40	14.7	2	5.4		42	13.6
Solid organ transplantation	47	17.3	12	32.4		59	19.1
Pulmonary disease	52	19.1	4	10.8		56	18.1
Other disease treated with steroids	10	3.7	0	0.0		10	3.2
Primary immunodeficiency	3	1.1	0	0.0		3	1.0
ICU^c^	44	16.2	6	16.2		50	16.2
COVID-19	48	17.6	6	16.2		54	17.5
Influenza	22	8.1	1	2.7		23	7.4
Other or unknown	22	8.1	2	5.4		24	7.8
**Underlying disease** ^d^							
At least one EORTC host factor	91	33.5	21	56.8	**0.012**	112	36.2
No EORTC host factor present	159	58.5	14	37.8		173	56.0

CAPA:  COVID-19-associated aspergillosis; EORTC: European Organisation for Research and Treatment of Cancer; IAPA:  influenza-associated aspergillosis; ICU:  intensive care unit; MSGERG: Mycoses Study Group Education and Research Consortium; NA:  not applicable.

^a^ Bold font is used to show p values indicating statistical significance.

^b^ NA: when more than one underlying risk factor was mentioned as possibly contributing (as several patients had more than one underlying factor).

^c^ Being in ICU was listed as ‘main underlying disease’ if no other underlying problem was reported; on the other hand, patients with COVID-19 or influenza were respectively listed as with such underlying diseases, even if they were in ICU.

^d^ A total of 24 patients with unknown main underlying disease were excluded. Host factor as defined by EORTC-MSGERC consensus definitions [15].

* p = 0.194: proven invasive aspergillosis classification vs other category classification (probable invasive aspergillosis, putative invasive aspergillosis, IAPA and CAPA).

^**^ p = 0.389: proven vs probable invasive aspergillosis.

^***^ p = 0.666: IAPA vs CAPA.

### Nearly 10% of the *Aspergillus fumigatus* isolates revealed decreased triazole susceptibility

**Figure 1:**
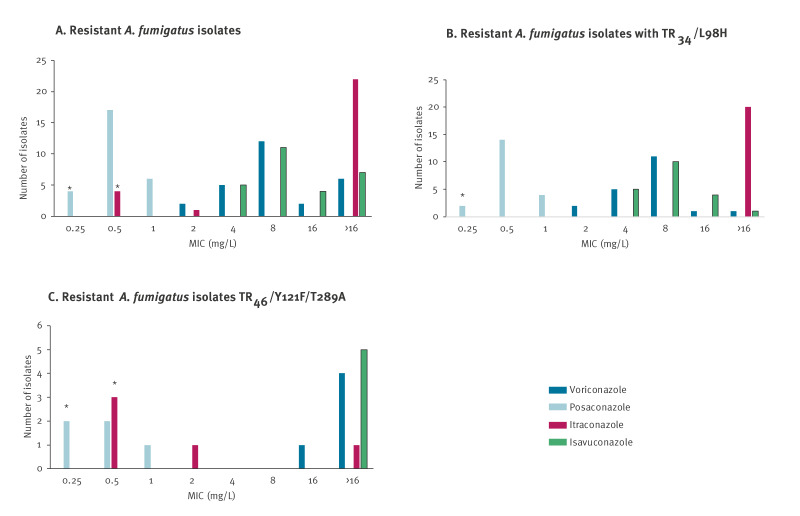
Detailed MIC values for four azoles^a^ tested for triazole-resistant *Aspergillus fumigatus* isolates (A) overall (n = 27), (B) with TR34/L98H (n = 20) and (C) with TR46/Y121F/T289A mutations (n = 5), Belgium, 1 April 2022–31 March 2023

EUCAST: European Committee on Antimicrobial Susceptibility Testing; MIC: minimum inhibitory concentration.

^a^ The four azoles were voriconazole, posaconazole, itraconazole and isavuconazole.

Triazole resistance screening by VIPCheck was performed for 278 *A. fumigatus sensu stricto* isolates from 275 patients, revealing the presence of decreased triazole susceptibility for 27 unique isolates (9.7%). Using EUCAST broth microdilution, all 27 isolates were confirmed to be resistant to at least two azoles, while all were susceptible to amphotericin B (MIC range: 0.25–1). Of these 27, 23 showed resistance to all four tested azoles, while the remaining isolates to three (2 isolates) and two (2 isolates) azoles respectively (Figure 1A). 

* In case the MIC value does not correspond to resistance according to EUCAST (v.10.0, 2020), MIC values > 1, > 0.25, > 1 and > 2 respectively for voriconazole, posaconazole, itraconazole and isavuconazole are interpreted as triazole resistant.

All isolates underwent *Cyp51A* gene sequencing, providing details on the resistance mechanism for 25 of 27 isolates, as for one isolate sequencing was repeatedly not successful and for the other no mutations were found. The TR_34_/L98H mechanism was the most prevalent (in 20 of the 26 successfully sequenced isolates) followed by TR_46_/Y121F/T289A (5/26) (Figures 1B and 1C). For TR_46_, additional mutations M172I and G448S were identified for one isolate. 

For the 31 non-*A. fumigatus* (sensu stricto) isolates, antifungal susceptibility was evaluated with EUCAST broth microdilution, revealing 10 resistant isolates, of which five isolates showed resistance to at least two of the four azoles tested, with only one conferring resistance to all four azoles. For three isolates elevated MIC values for amphotericin B were found. Details on the determined MIC50 and MIC90 values and their range are shown in Supplementary Table S2.

### High *Aspergillus fumigatus* triazole resistance rates in two provinces in Flanders

**Figure 2:**
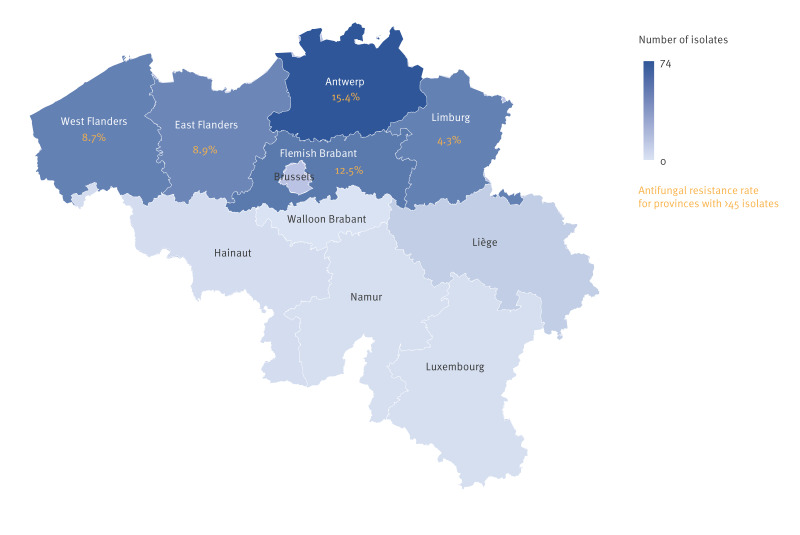
Number of *Aspergillus* isolates (n = 309) and triazole resistance rates for *Aspergillus fumigatus* isolates per province, Belgium, 1 April 2022−31 March 2023 (n = 278 *Aspergillus fumigatus* isolates)^a^

^a^ There were 278 *Aspergillus fumigatus isolates collected in Belgium, of which 250 originated from five Flemish provinces* (namely West-Flanders, East-Flanders, Antwerp, Limburg and Flemish Brabant).

Cases with resistant isolates were found to be characterised by a lower median age compared with susceptible isolates, 63 vs 67 years old (p = 0.033, Table 1). For the five provinces from where more than 45 isolates were sent, all five located in Flanders, large triazole resistance differences were observed for *A. fumigatus* isolates (Figure 2). In Antwerp, there were more isolates resistant compared to all other Flemish provinces (p = 0.082), and the proportion (15.4%; 10/65) was specifically higher than in the neighbouring province Limburg (2/46; 4.3%, p = 0.060).

The various levels in numbers of *Aspergillus* species complex isolates included in the study are represented on the map of Belgium, using different blue coloured shades. Provinces from which the isolates originated were deduced from postal codes of patients’ residence at the time of isolation of the fungus. Only the province of Walloon Brabant had no isolates included. For the five provinces from which more than 45 isolates originated (all five Flemish provinces in the northern part of the country, namely West-Flanders, East-Flanders, Antwerp, Limburg and Flemish Brabant), the percentages of triazole resistant *Aspergillus fumigatus* isolates are displayed on the respective mapped provinces. For the provinces of Liège, Luxembourg, Hainaut and Namur no resistant *A. fumigatus* isolates were detected, while for Brussels one isolate.

### Genetic relatedness of resistant *Aspergillus fumigatus* isolates

**Figure 3:**
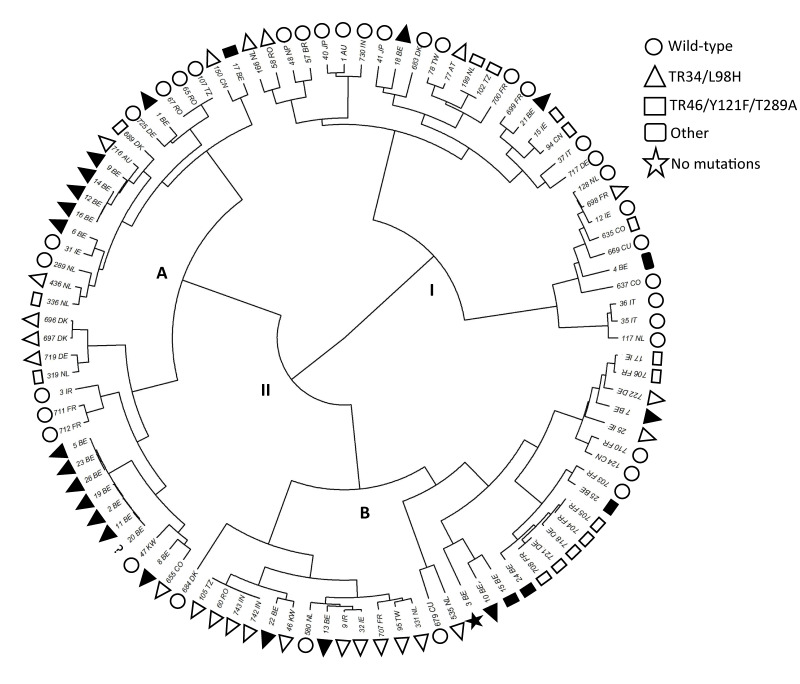
Circular phylogenetic tree depicting genetic relationship and characteristics of 26 triazole-resistant *Aspergillus fumigatus* study isolates according to short tandem repeats typing, Belgium, analysis in 2024 (n = 100 isolates analysed)

AU: Australia; AT: Austria; BR: Brazil; CN: China; CO: Colombia; CU: Cuba; DE: Germany; DK: Denmark; FR: France; I: India; IE: Ireland; IR: Iran; IT: Italy; JP: Japan; KW: Kuwait; NL: the Netherlands; NP: Nepal; RO: Romania; TW: Taiwan; TZ: Tanzania.

For 26 triazole-resistant *A. fumigatus* isolates, STR analysis was successful. Hierarchical clustering with 39 triazole-resistant and 35 susceptible global isolates showed that isolates are dispersed across two clades (Figure 3), and three Belgian isolates were in clade I whereas 23 were in clade II. Clade I mainly represents global susceptible isolates, while clade II triazole-resistant isolates. Clade II is divided into subclades A and B, with more Belgian isolates in subclade A (15 vs 8). Within subclade A, a cluster of seven very closely related Belgian isolates was identified, sampled across four provinces.

Characteristics and hierarchical clustering analysis (Euclidean distance and Ward’s minimum variance method) between triazole-resistant and selected susceptible isolates from our study and worldwide isolates describe two clades, clade I and clade II, with the latter divided in two subclades (A and B). Abbreviations are added for the origin of isolation, with the study isolates from Belgium coloured in black. The *Cyp51A* gene sequencing result is added to each isolate as specified in the legend. For one isolate, *Cyp51A* sequencing was not successful as depicted by the ‘?’ in the tree.

### Antifungal treatment of (resistant) invasive aspergillosis cases

Treatments started for the corresponding episodes of IA are represented in Supplementary Figure S2. For 88/297 patients (29.6%), either no antifungal treatment was started, or initiation was unknown. Considering 31 cases with triazole-resistant strains, who had therapy information available, three were started on therapy with in vitro activity against the *Aspergillus* species strain. In the remaining 28, first-line treatment was an azole to which the strain turned out to be resistant (Supplementary Figure S2). Antifungal treatment was more frequently changed for patients infected with a triazole-resistant *Aspergillus* species strain than for patients with a susceptible isolate (10 changes in 31 patients; 32.3% vs 16 changes in 178 patients; 9.0%, p = 0.0057; Supplementary Figure S2). Patients with resistant isolates were significantly more exposed to mould-active treatment within the year before infection compared with cases with susceptible isolates (8/37 vs 20/260, p < 0.02), as detailed in Supplementary Table S3.

## Discussion

During 1 year, 309 *Aspergillus* strains isolated from 297 cases diagnosed with IA were included in a nationwide surveillance study in Belgium. Triazole resistance was observed for 9.7% of *A. fumigatus* isolates, which corresponded to a doubling of the resistance rate over a period of 10 years (4.6% in 2011 [11]). Of *A. fumigatus* resistant strains, a majority (23 of 27) had reduced susceptibility to all four azoles investigated, with most (20 of 26) characterised by the *Cyp51A* resistance mechanism TR_34_/L98H. There appeared to be large geographical differences in triazole resistance rates in the country for *A. fumigatus*, with the highest rates in the provinces of Antwerp and Flemish Brabant. While therapy information was incomplete for an important share of the patients in our dataset, the first-line treatment in most patients with a triazole-resistant strain (28 of 31) was an azole, therefore inadequate and well-documented to be associated with increased mortality [23,24].

While triazole-resistant *A. fumigatus* isolates have been reported in all continents, resistance rates differ between countries, with the highest rates reported for Europe to date [25]. Highly variable triazole non-wild-type *A. fumigatus* rates, as observed in our study, were reported for Europe in a large-scale study conducted between 2017 and 2021 [26], advocating for the importance of nationwide surveillance programmes. Nearly reaching the threshold of 10% of triazole resistance in culture-positive cases, we are now left in Belgium with the option to switch current azole monotherapy schedules to combination therapies with echinocandins or liposomal amphotericin B, or complement with real-time PCR-based resistance testing, which may help to limit the clinical impact of triazole resistance [27]. In the 2017–2021 study [26], the *Cyp51A* TR_34_/L98H mutation was the most prevalent, in line with our findings and earlier studies [25,28]. This resistance mechanism has been reported to be selected in the environment [4], which stresses the need to address the growing problem of increasing incidence of triazole-resistance IA infections in a One Health context [29].

Agriculture, horticulture and composting facilities have been the subject of research on hotspots of triazole resistance selection in Europe [30], with no data available for Belgium until recently in 2024 [6]. Worldwide, up to one-third of total fungicide sales are azole agents, mostly triazoles, generally sprayed in fields to control mildew and rust affecting grass, grains, fruits, vegetables, and other crops [31,32]. Differences in local agriculture could potentially explain the uneven triazole resistance rates for *A. fumigatus* isolates between provinces in Flanders, with the rate in Antwerp (10/65) being almost 3.5 times that of neighbouring Limburg (2/46), even though both provinces are located near the border with the Netherlands. Greenhouse agriculture, including growing a large variety of fruits and vegetables, and associated auctions seem to be more concentrated in the province of Antwerp and West-Flanders according to the Flemish information centre for agriculture and horticulture [33]. Since environmental studies focusing on triazole resistance are nearly non-existing in Belgium [6], we can, however, only speculate about the regional differences in triazole resistance rates. It might also be noted that both provinces Antwerp and Flemish Brabant are more densely populated compared with Limburg. Moreover, the place where someone is domiciled is not necessarily the main location of environmental exposure as people travel, work or can have high professional exposure elsewhere. Although not significant and potentially biased due to the small sample size, the proportion of resistance appears smaller in Wallonia compared with Flanders. Walloon agriculture is characterised by a better developed organic farming sector and Wallonia generally has a lower level of urbanisation, providing more space for organic farming compared to Flanders (14% vs 2% of agricultural companies, according to the Ministry of Agriculture [34]).

As the proportion of patients previously exposed to azole therapy is significantly larger in triazole resistant aspergillosis, this shows that potentially not only environmental but also patient conditions play a role. Triazole resistant strains were characterised by a lower median age of the patient. This could reflect the fact that in a younger population a more intensive and longer treatment schedule for certain diseases is usually maintained. For non-fumigatus aspergillosis, resistant rates were higher (>30%), challenging antifungal treatment options, especially since elevated MIC values for amphotericin B were also observed for three in 10 cases. Only one case conferred resistance to all four azoles.

This study addresses triazole resistance in cases with *Aspergillus* positive cultures, while in clinical practice for more than half of cases this filamentous fungus does not grow from culture, and therefore PCR plays an important role to clinically assess azole resistance [29]. It would be of interest to also study cases of triazole resistance in aspergillosis identified by molecular testing in the future. An important limitation of the study is that the data at our disposal did not allow survival analysis, limiting our ability to link triazole resistance or inadequate first-line treatment to clinical outcomes. Secondly, limited clinical data were available for most strains that were sent by external laboratories, which posed challenges in drawing conclusions. Thirdly, unfortunately few isolates from Brussels and Wallonia were included, resulting in a predominance of isolates from Flanders in this study, limiting analyses on a more-refined geographical level to only Flemish provinces. In the study of 2011 [11], similarly, only two of the 18 laboratories that participated, were located in Wallonia. Overall, the considerable increase in triazole resistance rate in a 10-year period, stresses the importance of frequent and continued surveillance initiatives. Due to study participation, clinical laboratories are now more aware of triazole resistance and will hopefully lower their threshold to send clinical isolates of patients diagnosed with IA to the NRC for antifungal susceptibility testing and clinicians will be educated to adjust their treatment policy more quickly.

## Conclusions

Triazole resistance in patients with IA due to *A. fumigatus* in Belgium, doubled over a 10-year period with regional differences and rates > 10% in two densely populated provinces. Sustained and long-term national and international resistance surveillance data are warranted to guide treatment decisions as well as increased efforts to detect triazole resistance in clinical practice.

## Data Availability

Nucleotide sequencing data obtained within this study by performing *Cyp51A* gene sequencing and beta-tubulin gene sequencing were deposited to NCBI Genbank (PV563464−PV563499).

## Supplementary Data

376000 SupplementaryMaterial
