# Supplementary material for: Doubling of triazole resistance rates in invasive aspergillosis over a 10-year period, Belgium, 1 April 2022 to 31 March 2023
Source: Euro Surveill. 2025 May 8;30(18):2400559. doi: 10.2807/1560-7917.ES.2025.30.18.2400559 (PMC12066980; doi:10.2807/1560-7917.ES.2025.30.18.2400559)
Supplement: SupplementaryMaterial [file 2400559_SupplementaryMaterial.pdf]

This supplementary material is hosted by Eurosurveillance as supporting information alongside the article ‘Doubling of triazole resistance rates in invasive aspergillosis over a 10-year period, Belgium, 1 April 2022 to 31 March 2023’ on behalf of the authors, who remain responsible for the accuracy and appropriateness of the content. The same standards for ethics, copyright, attributions and permissions as for the article apply. Supplements are not edited by Eurosurveillance and the journal is not responsible for the maintenance of any links or email addresses provided therein.

Supplementary Figure S1. Overview of samples from which *Aspergillus* species strains were isolated.

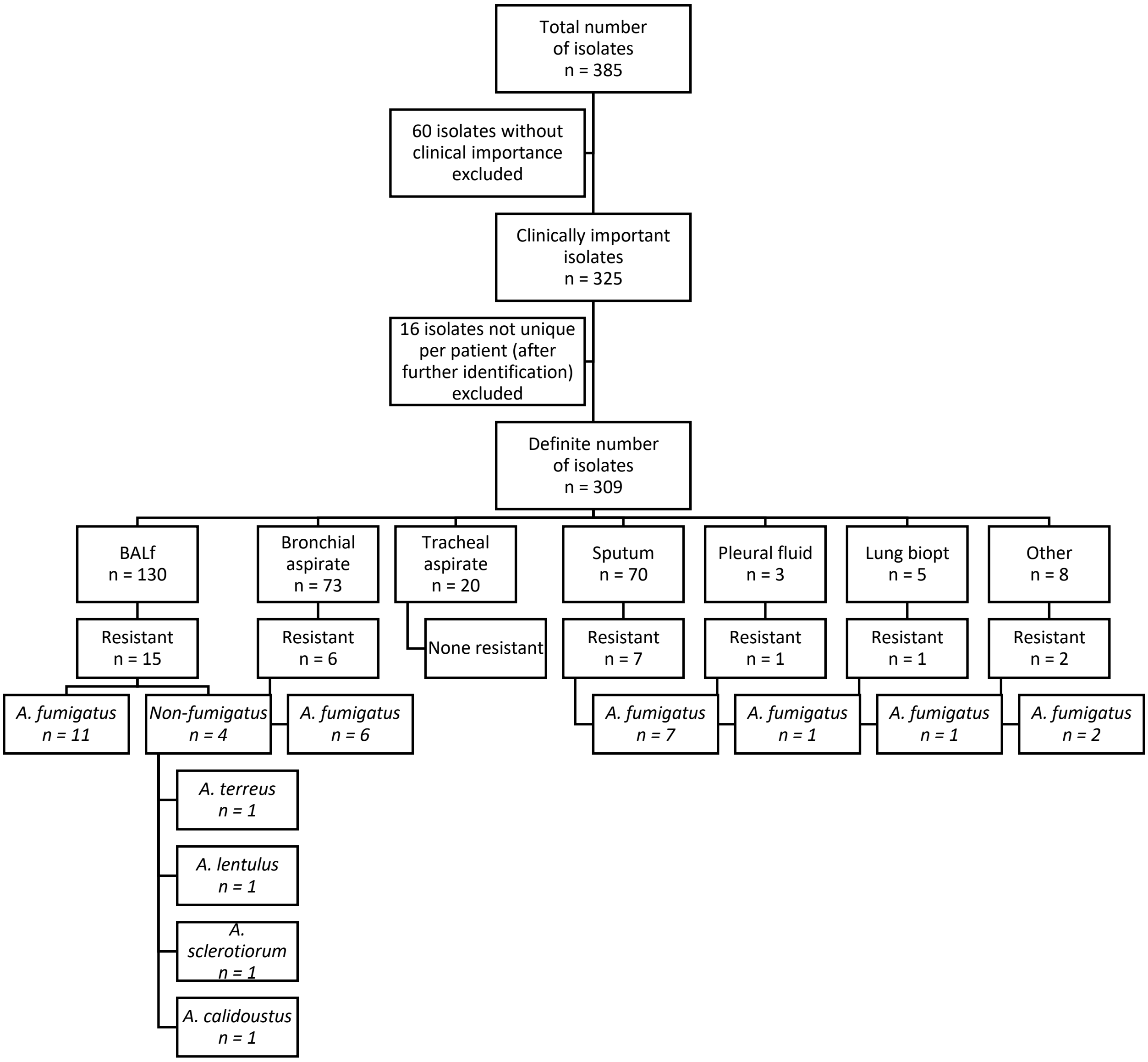

Supplementary Figure S2. First-, second- and third-line treatment started for episodes of invasive aspergillosis for patients includes in the study, Belgium (n = 297 patients).

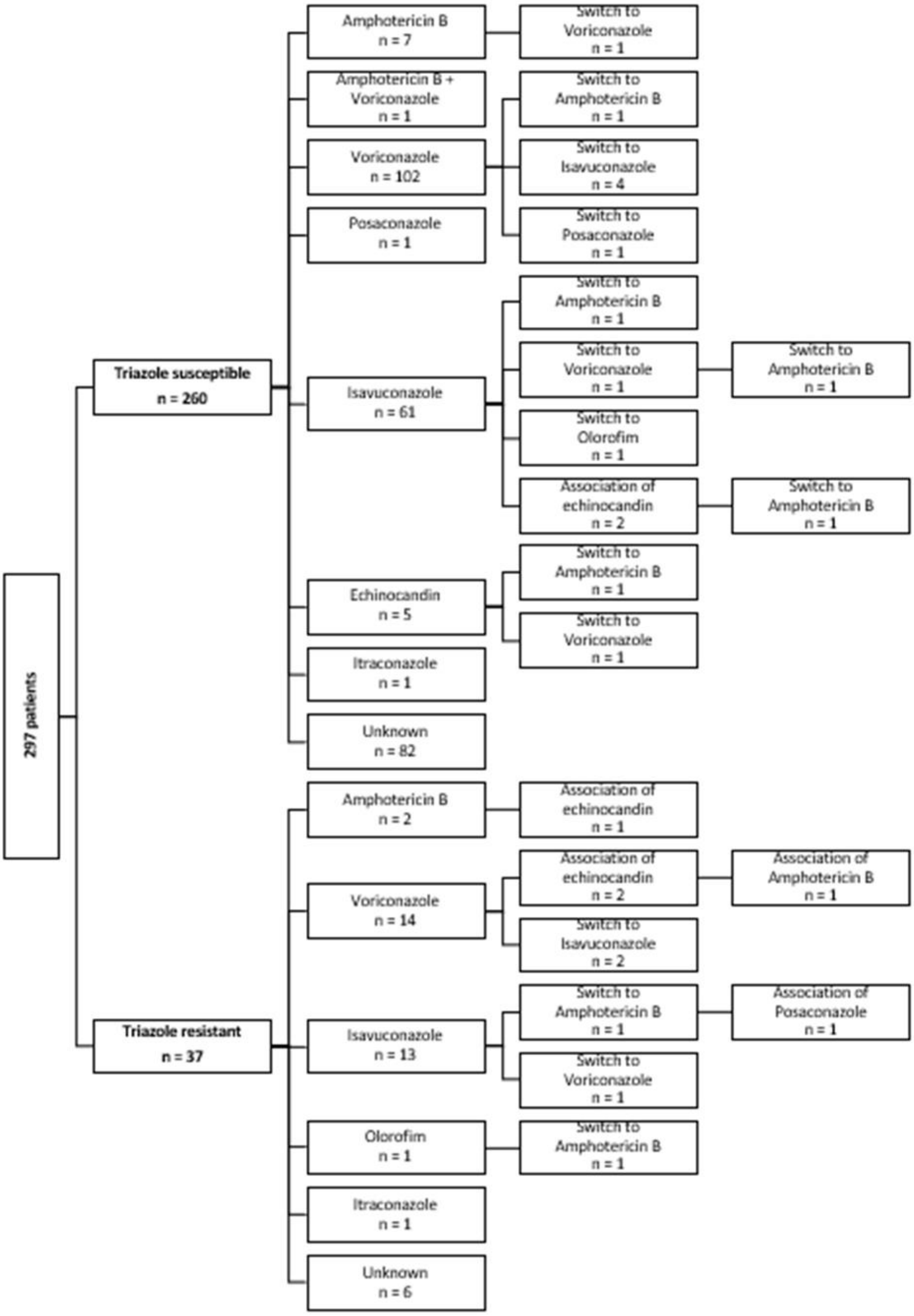

'Switch to' means that the antifungal treatment was changed during the course of the invasive aspergillosis episode, however not necessarily due to the detection of triazole resistance. 'Association of' means that an additional antifungal agent was added to the initial treatment as combination regimen.

Supplementary Table S1. Overview of resistance breakpoints applied to classify the isolates. Species-specific breakpoints as defined by EUCAST (2020, v10.0) were applied to all species within a same complex.[1] Epidemiological cut-off values (ECOFFs) as described in literature, were used to classify *Aspergillus* species isolates for which no EUCAST clinical breakpoints (2020, v10.0) are available. For species considered intrinsically resistant or not a good target for the drug, similar cut-off values were applied as for other species.

***Aspergillus* complex**	**MIC (mg/L) cutoff used for antifungal resistance (R>)**				
	**Amphotericin B**	**Voriconazole**	**Posaconazole**	**Itraconazole**	**Isavuconazole**
A. fumigatus	EUCAST v10.0				
A. niger	EUCAST v10.0	2 mg/L[1]	0.5 mg/L[1]	1 mg/L[1]	2 mg/L[1]
A. flavus	1 mg/L[1]	2 mg/L[1]	0.5 mg/L[1]	EUCAST v10.0	
A. nidulans	1 mg/L[1]	EUCAST v10.0	0.5 mg/L[1]	EUCAST v10.0	
A. terreus	1 mg/L[1]	2 mg/L[1]	EUCAST v10.0		
A. circumdatus	1 mg/L[1]	2 mg/L[1]	0.5 mg/L[1]	1 mg/L[1]	2 mg/L[1]
A. ustus	1 mg/L[1]	2 mg/L[1]	0.5 mg/L[1]	1 mg/L[1]	2 mg/L[1]

[1] Guinea J. Updated EUCAST clinical breakpoints against *Aspergillus*, implications for the clinical microbiology laboratory. J Fungi (Basel) (2020); 6(4): 343. https://doi.org/10.3390/jof6040343

Supplementary Table S2. Identification of 309 isolates to *Aspergillus* species level, in parallel to the MIC distribution (MIC50/MIC90 (range) in mg/L) of the evaluated antifungals, Belgium, 1 April 2022–31 March 2023 (n = 309 isolates)

Aspergillus	**Number**	**Proportion per complex %**	**MIC50/MIC90 (range)**				
			**Amphotericin B**	**Voriconazole**	**Posaconazole**	**Itraconazole**	**Isavuconazole**
***A. fumigatus* complex (n = 282; 91.3% of 309)**							
A. fumigatus	278	98.6	NA[a]				
A. lentulus	3	1.1	0.25/2 (0.25–2)	1/4 (1–4)	0.25/0.25 (0.125–0.25)	0.5/ > 16 (0.25– > 16)	1/4 (1–4)
A. brasiliensis	1	0.4	*0.25*	*2*	*0.25*	*1*	*4*
***A. niger* complex (n = 12; 3.9%)**							
A. tubingensis	7	58.3	0.125/0,125 (0.06–0.125)	1/2 (0.5–2)	0.25/0.25 (0.125–0.25)	0.5/1 (0.5–1)	2/4 (1–4)
A. welwitschiae	4	33.3	0.25/0;5 (0.125–0.5)	1/1 (0.5–1)	0.25/0.25 (0.125–0.25)	*0.5*	*2*
A. niger	1	8.3	*0.25*	*1*	*0.25*	*0.5*	*2*
***A. flavus* complex (n = 10; 3.2% of 309)**							
A. flavus	10	100	2/2 (0.25–4)	1/1 (1–8)	0.25/0.25 (0.125–0.25)	0.25/0.25 (0.125–0.25)	1/2 (1–4)
***A. nidulans* complex (n = 2; 0.6% of 309)**							
A. sublatus	1	50	*0.5*	*0.5*	*0.25*	*0.25*	*0.5*
A. quadrilineatus	1	50	*8*	*0.125*	*0.06*	*0.06*	*0.06*
***A. terreus* complex (n = 1; 0.3% of 309)**							
A. terreus	1	100	*4*	*2*	*0.06*	*0.25*	*2*
***A. circumdatus* complex (n = 1; 0.3% of 309)**							
A. sclerotiorum	1	100	*> 16*	*4*	*0.5*	*4*	*4*

***A. ustus* complex (n = 1; 0.3% of 309)**							
A. calidoustus	1	100	*0.5*	*8*	*> 8*	*> 16*	*4*

EUCAST: European Committee on Antimicrobial Susceptibility Testing; MIC: minimum inhibitory concentration; NA: not applicable.

[a] For *Aspergillus fumigatus* isolates, MICs are only available when the first triazole resistance screening (VIPCheck) showed reduced susceptibility, this is why they are not listed.

The actual MIC values as determined by the EUCAST broth microdilution method are listed in italics, when only one isolate was available for a particular *Aspergillus* species, or when the same MIC values were observed for the different isolates of the same *Aspergillus* species (e.g. MIC value of 0.5 for itraconazole for all four *A. welwitschiae* isolates).

Supplementary Table S3. Overview of mould-active treatment within the year before infection (when available).

Previous mould-active therapy (≤1 year)	Number of patients n = 28
Amphotericin B	1
Voriconazole	12
Posaconazole	2
Isavuconazole	4
Voriconazole and Posaconazole	1
Voriconazole and Isavuconazole	3
Posaconazole and Isavuconazole	1
Itraconazole	2
Voriconazole, Posaconazole and Amphotericin B	1
Voriconazole, Isavuconazole and Amphotericin B	1
